# Neoadjuvant chemotherapy may be the best neoadjuvant therapy modality for non-metastatic pancreatic cancer: a population based study

**DOI:** 10.3389/fonc.2024.1370009

**Published:** 2024-04-10

**Authors:** Jie Yang, Xiang Qu, Fan Jiang, Hong-mei Qiao, Jie Zhao, Jin-ru Zhang, Li-juan Yan, An-jie Zheng, Peng Ning

**Affiliations:** Department of Oncology, Baoji Gaoxin Hospital, Baoji, China

**Keywords:** pancreatic cancer, neoadjuvant therapy, neoadjuvant chemotherapy, neoadjuvant chemoradiotherapy, prognosis

## Abstract

**Objective:**

Currently, there are no studies showing which neoadjuvant therapy modality can provide better prognosis for patients after pancreatic cancer surgery. This study explores the optimal neoadjuvant therapy model by comparing the survival differences between patients with non-metastatic pancreatic cancer (cT1-4N0-1M0) who received neoadjuvant chemotherapy (NACT) and neoadjuvant chemoradiotherapy (NARCT).

**Methods:**

We retrospectively analyzed the clinical data of 723 patients with cT1-4N0-1M0 pancreatic cancer who received neoadjuvant therapy before surgery from the Surveillance, Epidemiology, and End Results (SEER) database. After propensity score matching (PSM), we compared the effects of NACT and NARCT on overall survival (OS) and cancer-specific survival (CSS) in patients with non-metastatic pancreatic cancer, and then performed subgroup analyze. Finally, we used univariate and multivariate Cox regression analysis to explore potential risk factors for OS and CSS in patients with non-metastatic pancreatic cancer treated with preoperative neoadjuvant therapy.

**Result:**

Before PSM, mOS (30.0 months VS 26.0 months, P=0.122) and mCSS (30.0 months VS 26.0 months, P=0.117) were better in patients with non-metastatic pancreatic cancer treated with NACT compared with NARCT, but this was not statistically significant (P>0.05). After PSM, mOS (30.0 months VS 25.0 months, P=0.032) and mCSS (33.0 months VS 26.0 months, P=0.028) were better in patients with non-metastatic pancreatic cancer treated with NACT compared with NARCT, and this difference was statistically significant (P<0.05). Multivariate Cox regression analysis results showed that age, lymph node positivity, and NARCT were independent adverse prognostic factors for OS and CSS in patients with non-metastatic pancreatic cancer.

**Conclusion:**

The study results show that compared with NARCT, NACT is the best preoperative neoadjuvant therapy mode for patients with non-metastatic pancreatic cancer. This result still needs to be confirmed by more prospective randomized controlled trials.

## Introduction

Pancreatic cancer is a highly aggressive malignant tumor of the digestive tract with a five-year survival rate of only 10%, which has increased by only 5% over the past 20 years (1). Surgery and postoperative adjuvant chemotherapy (ACT) have always been the standard treatments for pancreatic cancer, and past research mainly focused on ACT (2–4). In order to improve the survival of patients with pancreatic cancer, it is necessary to conduct multimodal research. Neoadjuvant chemotherapy (NACT) with or without radiotherapy is increasingly used in patients with pancreatic cancer. This may be related to the fact that patients who plan to undergo surgery often have local unresectable lesions or micro-metastases that cannot be predicted by imaging during surgical exploration. In the end, only 20% of patients underwent surgical resection. The second reason may be that less than 60% of patients can tolerate systemic therapy after surgery (5), and about 50% of patients who do not receive ACT will relapse within half a year (2). Neoadjuvant therapy can increase the opportunity for patients with pancreatic cancer to receive systemic therapy, increase the R0 resection rate, and improve the overall survival (OS) of patients (6, 7). A Meta analysis that included six randomized controlled trials showed that neoadjuvant therapy significantly improved OS (HR= 0.73, 95%CI: 0.61-0.86) in patients with pancreatic cancer (8). However, it is unclear which neoadjuvant therapy modality is optimal.

The Italian PACT-5 study found that neoadjuvant chemotherapy can reduce the 1-year event-free survival (EFS) rate of resectable pancreatic cancer (RPC) compared with primary surgery (9). The Prep-02/JSAP05 study also found that the combination of gemcitabine and S1 neoadjuvant therapy improved survival by about 10 months compared to direct postoperative S1 adjuvant chemotherapy (10). Two randomized controlled studies have shown that neoadjuvant chemoradiotherapy (NARCT) can improve the OS of patients with pancreatic cancer compared with direct surgery (11–13). However, the results of the phase II A021501 randomized controlled trial showed (14) that compared with 8 cycles of neoadjuvant mF0LFIRINOX chemotherapy regimen, 7 cycles of neoadjuvant mF0LFIRINOX chemotherapy plus SBRT in patients with borderline resectable pancreatic cancer (BRPC) had shorter median EFS (10.2 months VS 15.0 months) and mOS (17.1 months VS 29.8months), and R0 resection rate (33% VS 57%) is lower. This finding raises the question of whether additional radiation therapy may reduce the effectiveness of ACT.

Currently, there are few studies comparing the impact of different neoadjuvant therapy modalities on the survival of patients with pancreatic cancer. Therefore, in this retrospective analysis based on the Surveillance, Epidemiology, and End Results (SEER) database, this study evaluated the difference between NARCT and NACT in improving survival in patients with pancreatic cancer who underwent surgical treatment. Furthermore, we used propensity score matching (PSM) analysis to reduce the impact of confounding factors in non-randomized controlled studies. At the same time, we performed subgroup analysis of patients to determine the most appropriate neoadjuvant therapy mode for each subgroup.

## Method

### Patient characteristics

**Figure 1:**
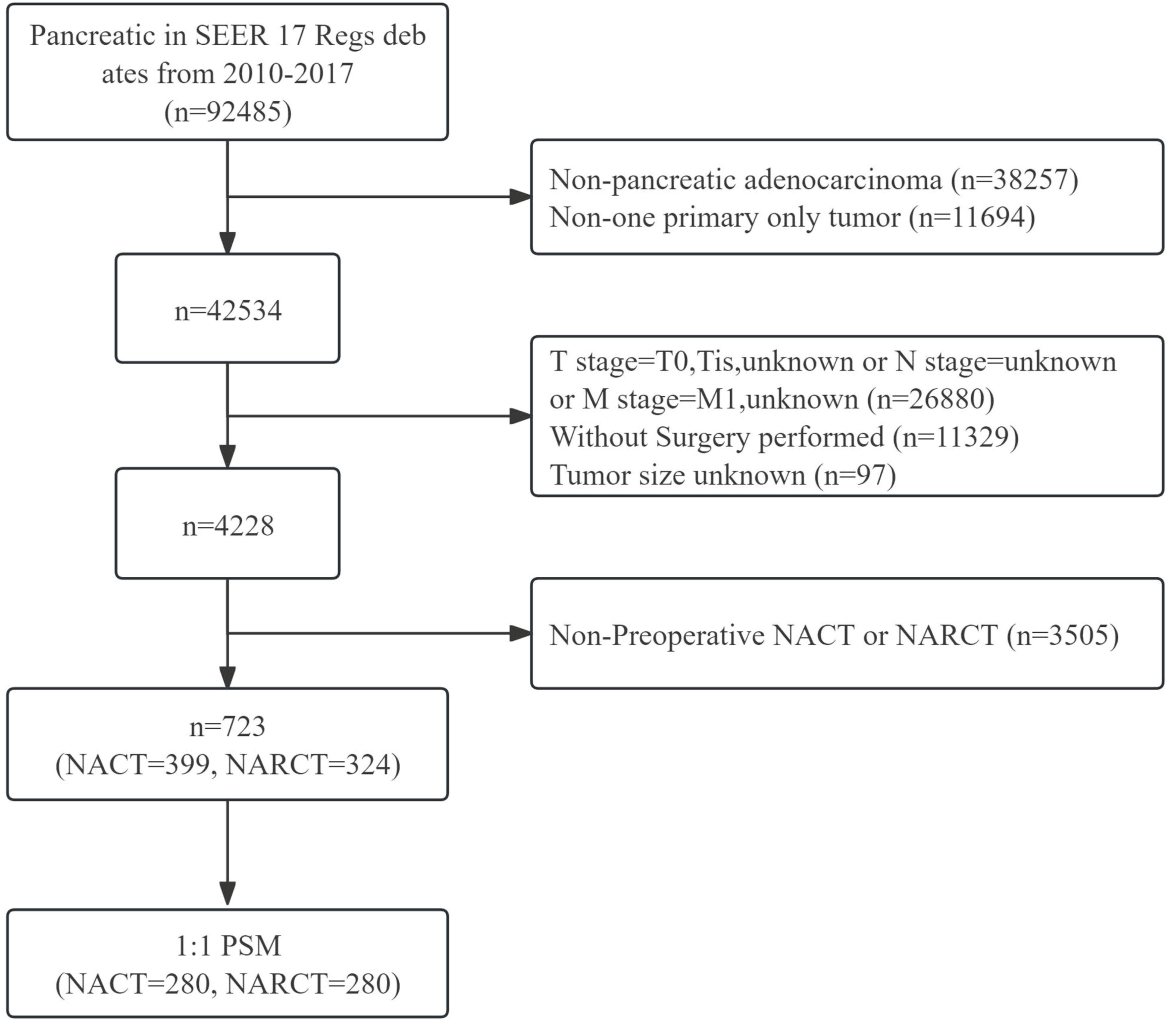
Flow chart of inclusion and exclusion of pancreatic cancer patients receiving neoadjuvant therapy from 2010 to 2017.

The data source for this study is the SEER database. The SEER database is a public cancer registry maintained by the National Cancer Institute (NCI) in the United States. Our study retrospectively analyzed the survival outcomes of patients with non-metastatic pancreatic cancer (cT1-4N0-1M0) who received different neoadjuvant therapies in the SEER database. We included patients with surgically resected non-metastatic pancreatic cancer from 2010 to 2017, among whom patients who received NACT or NARCT before surgery were included in the study. The histological type of the subjects studied in this study was pancreatic adenocarcinoma, the ICD-03 histological/behavioral code was 8140/3 (adenocarcinoma), and there were no other primary tumors. We collected important clinical characteristics, such as age, sex, race, primary site, tumor size, tumor differentiation, tumor stage, T stage, lymph node status, type of neoadjuvant therapy and whether to receive ACT after surgery. While the SEER database does not provide detailed information on neoadjuvant therapy, it extensively documents the occurrence and sequence of interventions such as surgery, radiotherapy, and chemotherapy. Considering this, our study defines chemotherapy administered before surgery as NACT. Additionally, regimens involving both chemotherapy and radiotherapy prior to surgery are categorized as NARCT, with the radiotherapy component being exclusively external beam radiotherapy. This study ultimately enrolled 723 patients with non-metastatic pancreatic cancer who received neoadjuvant therapy **Figure 1**.

### Statistical analysis

The chi-square test was used to compare the differences in clinical and pathological characteristics between the NACT and NARCT groups. PSM is a statistical method that improves the credibility of research results by controlling selection bias in observational studies. PSM was employed to balance the two groups of clinical and pathological features using a 1:1 ratio, nearest neighbor matching, and a caliper of 0.2. The Kaplan-Meier method was used to estimate OS and CSS in the two groups, and the log-rank test was used to compare the survival differences. Single-factor and multi-factor Cox regression models were used to study the risk factors of OS and CSS. Variables with P ≤ 0.05 in univariate analysis were subsequently included in multivariate analysis. All statistical analyses were performed using R statistical software version 4.2.2. Bilateral P<0.05 was considered statistically significant.

## Results

### Patient characteristics

From 2010 to 2017, among patients with cT1-4N0-1M0 pancreatic cancer registered in the SEER database, a total of 723 patients with pancreatic cancer received neoadjuvant therapy followed by surgery. Among these patients, 399 received NACT and 324 received NARCT. The majority of patients were aged 55 to 69 years (57.4%), white (86.6%), pancreatic head cancer (77.0%), stage II (70.7%), T3 (65.8%), and tumor size 2 to 4cm (63.8%). It is worth noting that more patients who received NARCT were stage III, T4, with tumor size >4cm and did not receive ACT after surgery. There was no significant difference in sex, race, tumor location and Pathological grade between the two groups (P> 0.05). 1:1 PSM was used to balance the differences between the two groups, and the final matching sample was 280 patients who received preoperative NACT. There was no statistical difference in the baseline pathological characteristics between the two groups after matching (P>0.05). The baseline pathological characteristics of patients before and after PSM are shown in **Table 1**.

**Table 1:** Baseline characteristics of patients included in the analysis before and after PSM.

Characteristic	Before PSM			After PSM		
	NACT (N, %)	NARCT (N, %)	*P*	NACT (N, %)	NARCT (N, %)	*P*
Age						
30-54	82(20.6)	64 (19.8)	0.006	56(20.0)	52(18.6)	0.199
55-69	211(52.9)	204 (63.0)		161(57.5)	180(64.3)	
≥70	106(26.6)	56(17.3)		63(22.5)	48(17.1)	
Sex						
Female	203(50.9)	163(50.3)	0.938	137(48.9)	145(51.8)	0.554
male	196(49.1)	161(49.7)		143(51.1)	135(48.2)	
Race						
White	347(87.0)	279(86.1)	0.821	244(87.1)	240(85.7)	0.711
other	52(13.0)	45(13.9)		36(12.9)	40(14.3)	
Primary						
Head	308(77.2)	249(76.9)	0.454	220(78.6)	222(79.3)	0.966
Body/tail	47(11.8)	46(14.2)		34(12.1)	32(11.4)	
Other	44(11.0)	29(9.0)		26(9.3)	26(9.3)	
Grade						
1	22(5.5)	26(8.0)	0.365	20(7.1)	23(8.2)	0.957
2	103(25.8)	75(23.1)		64(22.9)	67(23.9)	
3	78(19.5)	51(15.7)		49(17.5)	45(16.1)	
4	3(0.8)	2(0.6)		3(1.1)	2(0.7)	
Unknown	193(48.4)	170(52.5)		144(51.4)	143(51.1)	
Stage						
I	43(10.8)	31(9.6)	<0.001	26(9.3)	30(10.7)	0.853
II	301(75.4)	210(64.8)		202(72.1)	199(71.1)	
III	55(13.8)	83(25.6)		52(18.6)	51(18.2)	
T						
1	19(4.8)	4(1.2)	<0.001	5(1.8)	4(1.4)	0.736
2	50(12.5)	35(10.8)		27(9.6)	35(12.5)	
3	276(69.2)	200(61.7)		197(70.4)	190(67.9)	
4	54(13.5)	85(26.2)		51(18.2)	51(18.2)	
N						
0	176(44.1)	174(53.7)	0.013	138(50.3)	140(50.0)	0.933
1	223(55.9)	150(46.3)		142(50.7)	140(50.0)	
Tumor Size						
≤2cm	58(14.5)	17(5.2)	<0.001	17(6.1)	17(6.1)	0.740
2-4cm	259(64.9)	202(62.3)		185(66.1)	193(68.9)	
>4cm	82(20.6)	105(32.4)		78(27.9)	70(25.0)	
ACT						
No	266(66.6)	245(75.6)	0.011	201(71.8)	205(73.2)	0.776
Yes	133(33.3)	79(24.4)		79(28.2)	75(26.8)	

### Survival before PSM

**Figure 2:**
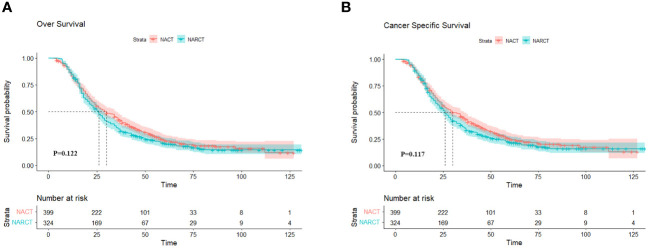
OS **(A)** and CSS **(B)** of two groups of NACT and NARCT before PSM.

The median follow-up time for all patients who met the criteria was 27.0 months. (IQR 16,47). **Figures 2A**, **B** show the Kaplan-Meier curves of OS and cancer-specific survival (CSS) of the two groups of patients respectively. Log-rank test results showed that NARCT appeared to be associated with worse mOS (26.0 months VS 30.0 months, P=0.122) and mCSS (26.0 months VS 30.0 months, P=0.117) compared with NACT, although this result was not statistically different (P >0.05).

### Survival after PSM

**Figure 3:**
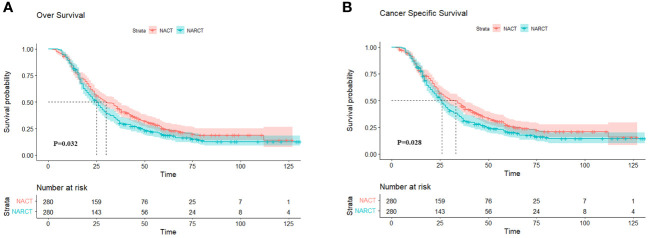
OS **(A)** and CSS **(B)** of two groups of NACT and NARCT after PSM.

After PSM, OS was worse in the NARCT group compared with NACT (P= 0.032) (**Figure 3A**). The mOS was 30.0 months (95%CI: 24.58-35.42) in the NACT group and 25.0 months (95%CI: 22.05-27.95) in the NARCT group. The log-rank test showed significant differences in CSS between the two groups (*P*= 0.028) (**Figure 3B**). Moreover, the mCSS of the NACT group was higher than that of the NARCT group, with the mCSS of the two groups of patients being 33.0 months (95%CI: 27.30-38.70) and 26.0 months (95%CI: 23.19-28.81) respectively.

**Figure 4:**
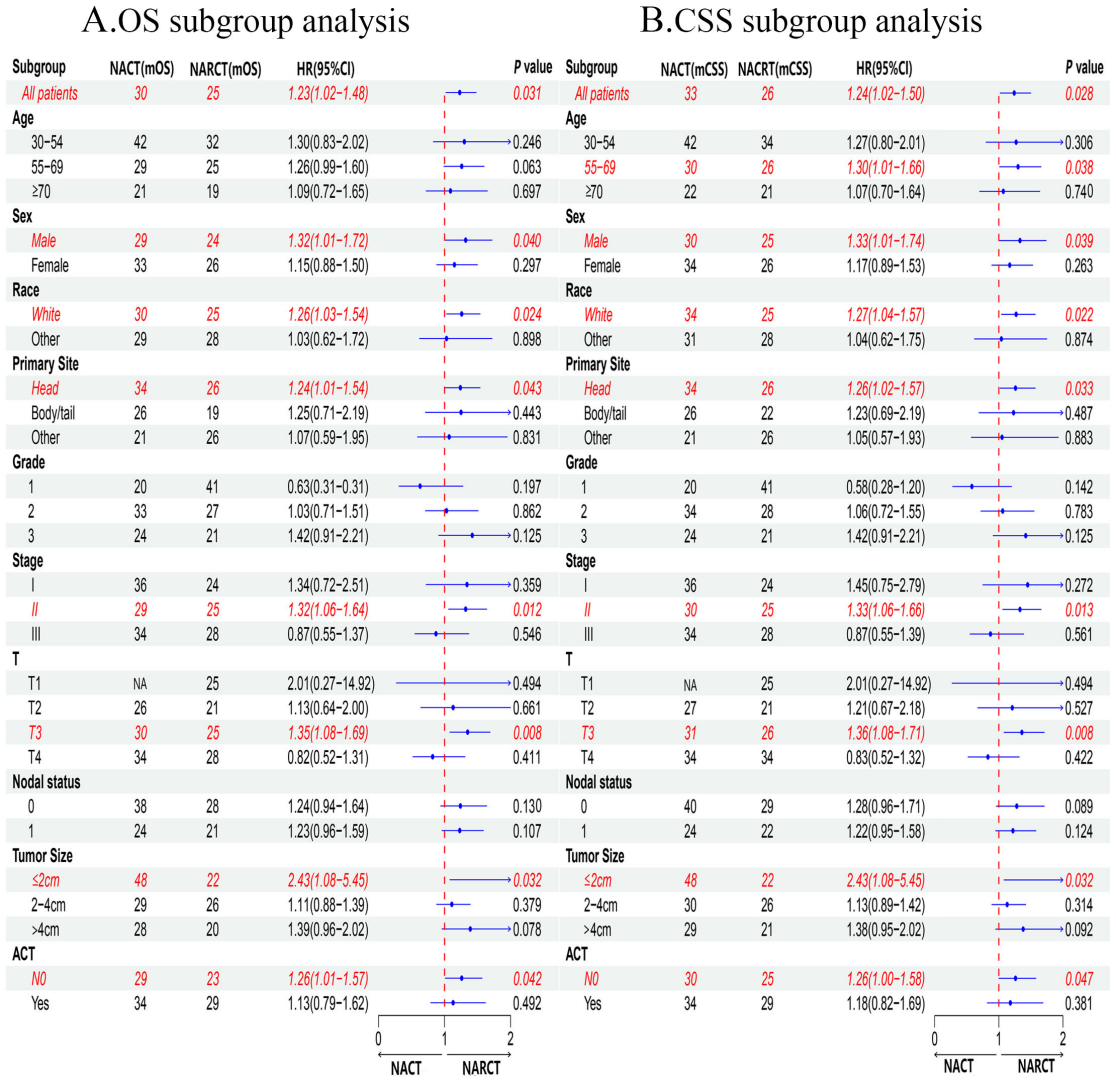
Subgroup analysis of OS **(A)** and CSS **(B)** after PSM.

In addition, our subgroup analysis results (**Figure 4**) based on matching clinical and pathological characteristics of patients showed that, regardless of OS (**Figure 4A**) or CSS (**Figure 4B**), for male, white, pancreatic head cancer, stage II, T3, tumor ≤2cm, the prognosis of patients receiving NARCT is worse than that of NACT. In the 55-69 years old subgroup, we found that NARCT was a risk factor for the prognosis of CSS. It is worth noting that in most other subgroups, the NACT group was better than the NARCT group in terms of OS and CSS, although this result was not statistically significant (P> 0.05).

**Table 2** shows the prognostic factors associated with pancreatic cancer OS in univariate and multivariate Cox regression analysis after PSM. Univariate Cox regression analysis showed that lower OS of pancreatic cancer was related to age, lymph node metastasis, and NARCT. In multivariate Cox analysis, these factors were still independent factors significantly associated with lower OS in pancreatic cancer.

**Table 2:** Univariate and multivariate analysis of OS for the matched cohort after PSM.

Characteristics	Univariate			Multivariate		
	HR	CI	*P*	HR	CI	*P*
Age						
30-54	Reference			Reference		
55-69	1.30	1.01 - 1.66	0.043	1.31	1.02 - 1.68	0.035
≥70	1.61	1.19 - 2.18	0.002	1.62	1.20 - 2.20	0.002
Sex						
Male	Reference					
Female	0.89	0.74 - 1.07	0.227			
Race						
White	Reference					
Other	0.98	0.75 - 1.29	0.913			
Primary						
Head	Reference					
Body/tail	1.04	0.77 - 1.40	0.793			
Other	1.12	0.82 - 1.54	0.477			
Grade						
1	Reference					
2	1.17	0.79 - 1.74	0.427			
3	1.48	0.98 - 2.24	0.060			
4	1.87	0.66 - 5.29	0.240			
Stage						
I	Reference					
II	1.33	0.95 - 1.84	0.093			
III	1.08	0.74 - 1.59	0.694			
T						
1	Reference					
2	2.29	0.83 - 6.34	0.112			
3	2.37	0.88 - 6.36	0.086			
4	1.98	0.72 - 5.42	0.184			
N						
0	Reference			Reference		
1	1.50	1.24 - 1.81	<0.001	1.51	1.25 - 1.83	<0.001
Tumor Size						
≤2cm	Reference					
2-4cm	1.23	0.82 - 1.85	0.319			
>4cm	1.24	0.81 - 1.92	0.322			
ACT						
No	Reference					
Yes	0.90	0.73 - 1.11	0.319			
Neoadjuvant therapy						
NACT	Reference			Reference		
NARCT	1.23	1.02 - 1.48	0.032	1.25	1.04 - 1.51	0.019

**Table 3** shows the prognostic factors associated with CSS of pancreatic cancer in univariate and multivariate Cox regression analysis after PSM. Univariate Cox regression analysis showed that lower CSS in pancreatic cancer was associated with age, Grade 3, lymph node positivity, and NARCT. In multivariate Cox regression analysis, we found that these factors remained independent factors significantly associated with lower CSS in pancreatic cancer.

**Table 3:** Univariate and multivariate analysis of CSS for the matched cohort after PSM.

Characteristics	Univariate			Multivariate		
	HR	CI	*P*	HR	CI	*P*
Age						
30-54	Reference			Reference		
55-69	1.33	1.02 - 1.72	0.033	1.36	1.05 - 1.77	0.019
≥70	1.66	1.22 - 2.27	0.001	1.71	1.25 - 2.34	<0.001
Sex						
Male	Reference					
Female	0.90	0.74 - 1.09	0.270			
Race						
White	Reference					
Other	1.00	0.76 - 1.32	0.997			
Primary						
Head	Reference					
Body/tail	1.02	0.75 - 1.38	0.906			
Other	1.12	0.81 - 1.54	0.510			
Grade						
1	Reference			Reference		
2	1.22	0.81 - 1.83	0.347	1.37	0.91 - 2.07	0.130
3	1.58	1.04 - 2.41	0.033	1.72	1.13 - 2.63	0.012
4	1.50	0.46 - 4.92	0.504	1.49	0.45 - 4.92	0.516
Stage						
I	Reference					
II	1.37	0.97 - 1.92	0.073			
III	1.13	0.76 - 1.68	0.542			
T						
1	Reference					
2	2.15	0.77 - 5.96	0.143			
3	2.26	0.84 - 6.05	0.106			
4	1.92	0.70 - 5.25	0.206			
N						
0	Reference			Reference		
1	1.57	1.30 - 1.91	<0.001	1.59	1.31 - 1.92	<0.001
Tumor Size						
≤2cm	Reference					
2-4cm	1.17	0.78 - 1.76	0.447			
>4cm	1.19	0.77 - 1.84	0.435			
ACT						
No	Reference					
Yes	0.91	0.74 - 1.13	0.399			
Neoadjuvant therapy						
NACT	Reference			Reference		
NARCT	1.24	1.02 - 1.50	0.028	1.30	1.07 - 1.58	0.008

## Discussion

Pancreatic cancer is a malignant tumor with high mortality. It is estimated that by 2030, it will become the second leading cause of cancer death in the United States (15). Due to the fact that most patients with pancreatic cancer have lost the opportunity for surgery at the time of diagnosis, and the completion rate of postoperative chemotherapy is also low, neoadjuvant therapy has become an important means to improve patient survival of pancreatic cancer.

Our study found that before PSM, patients with pancreatic cancer who received NACT had a trend of OS and CSS benefit compared with NARCT, while after PSM, patients with non-metastatic pancreatic cancer who received NACT had significant OS and CSS benefit. Subgroup analysis showed that compared with NARCT, OS and CSS of pancreatic cancer patients receiving NACT had significant benefits in male, white, pancreatic head cancer, stage II, T3, tumor size ≤ 2cm, and non-ACT subgroups. At the same time, in other subgroups, patients receiving NACT have a tendency to have better OS and CSS than pancreatic cancer patients receiving NARCT. Multivariate COX regression analysis confirmed that NACT was an independent prognostic risk factor for OS and CSS in patients with non-metastatic pancreatic cancer. Our findings are consistent with the A021501 study (14) and support NACT as the preferred neoadjuvant therapy strategy for patients with non-metastatic pancreatic cancer.

An increasing number of studies support the application of neoadjuvant therapy, which can convert patients with originally inoperable non-metastatic pancreatic cancer into resectable patients, thereby providing the possibility of curative surgery and improved survival. At the same time, neoadjuvant therapy can also screen out those patients who can really benefit from surgery and avoid ineffective surgery for patients whose disease progresses during neoadjuvant therapy. This may be due to the fact that the tumors in these patients are too aggressive and surgery cannot bring benefits (16–19). Although radiotherapy can further improve the surgical resection rate of patients on the basis of chemotherapy (18, 20, 21), whether this can improve patient survival is controversial. NARCT is associated with a higher surgical resection rate for pancreatic cancer (21). More than half of inoperable locally advanced pancreatic cancer (LAPC) can be converted to surgical resection. Compared with patients without surgery, patients after surgical resection have a significant survival benefit (17) (15.3 months VS 8.5 months, P<0.001). The single-arm LAP1 study (22) showed that compared with historical controls, NARCT improved the survival rate of patients with LAPC. 18% (7/39) of patients were converted to operable and all underwent R0 resection. The median OS of patients in the resection group was better than Unresected group (24.0 months VS 15 months, *P*= 0.03).

The phase III CONKO-007 study (23) found that compared to NACT, although NARCT can improve the negative circumferential margin rate and pathological complete response (pCR) rate in patients with LAPC, the primary endpoint of both groups was R0 There was no significant difference in resection rates, and this resectability effect was not associated with a progression-free survival (PFS) or OS benefit. Another phase III LAP07 study (24) found that although NARCT could improve the local control rate of LAPC compared with induction chemotherapy, this did not translate into an OS benefit (15.2 months VS 16.5 months, P= 0.83). The PREOPANC long-term follow-up study (12) found that NARCT can significantly improve the 5-year OS rate of patients with RPC, but the results of this study are also somewhat controversial. Firstly, for patients with RPC, there is no significant difference in OS between NARCT and primary surgery (HR= 0.79, 95% CI: 0.54-1.16, P= 0.23). Secondly, NARCT only prolonged mOS by 1.4 months, and at baseline, more patients in the NARCT group had a WHO performance status score of 0 (58% VS 39%), fewer patients were over 65 years old (54% VS 65%), and fewer patients had tumors in the pancreatic head (83% VS 92%). Additionally, a smaller proportion of patients had CA19-9 levels greater than 500 U/ml (29% vs. 35%). These factors may have affected the comparability of the two groups of patients, making interpretation of the results more difficult. The PREOPANC-2 study (25) found that FOLFIRINOX-based NACT and gemcitabine-based NARCT were similar in terms of surgical resection rate and OS for RPC and BRPC, indicating that radiotherapy may make up for the shortcomings of gemcitabine because FOLFIRINOX is more effective than gemcitabine alone (26, 27). In the phase II randomized controlled A021501 study (14), both NARCT and NACT were based on the same mFOLFIRINOX regimen, excluding the impact of differences in the effectiveness of different chemotherapy regimens. The results show that additional radiotherapy is a factor for the poor prognosis of pancreatic cancer, and this is not due to the increased toxicity caused by radiotherapy. The possible explanation for this result is that the mFOLFIRINOX treatment delay rate and dose reduction rate were higher in the chemoradiotherapy group, which may lead to a decrease or disappearance of treatment efficacy for systemic diseases.

Improvements in radiotherapy strategies, such as SBRT, may be the focus of neoadjuvant therapy of pancreatic cancer in the future. The advantages of SBRT are short treatment time, reduced risk of chemotherapy interruption, and higher biologically effective dose (BED) that can increase local tumor control rate and translate into survival benefit (14, 20, 28). Studies have shown that BED greater than 70Gy is associated with better survival benefit in pancreatic cancer (29, 30). In the A021501 study (14), the maximum BED (55Gy) of SBRT was far lower than the ablation dose required for SBRT, which may be the reason for the poor efficacy of the SBRT group. Improved radiotherapy technology, such as Isotoxic High-Dose Stereotactic Body Radiotherapy (iHD-SBRT), can individually adjust the radiotherapy dose and fractionation times to maximize the killing of tumor cells and minimize damage to surrounding normal tissues (28, 31). In addition, combined radiotherapy and immunotherapy may be a potential therapeutic strategy, although the effects of immunotherapy alone are limited in pancreatic cancer due to the characteristics of low tumor mutation load and immunosuppressive microenvironment (32, 33). However, in a phase II study (34) involving 29 patients, the efficacy of tislelizumab combined with AG-based concurrent chemoradiotherapy as neoadjuvant therapy for BRPC and LAPC was evaluated. The objective response rate was 60%, the R0 resection rate was 90% (9/10), and the 1-year OS rate was 72%. This suggests that radiotherapy can synergize with immunotherapy to enhance the immune response of tumors.

### Limitations

Although the SEER database collects a large number of patient groups and long-term survival data, it also has some important limitations. Firstly, Compared with SEER’s huge cancer data, our sample size is still small. Secondly, because it is a retrospective study, even if we used PSM to reduce selection bias, bias is still inevitable. Thirdly, the SEER database lacks some key variables, such as specific chemotherapy regimens, radiotherapy techniques, target volume design and dose, as well as confounding factors that may affect prognosis (eg: resectability status, surgical margin status, health status, surgical complications disease, serum CA19-9 levels and ECOG performance status, et.). Finally, although mutations in homologous genes BRCA1/BRCA2 increase the risk of pancreatic cancer, they also appear to be associated with better neoadjuvant efficacy, but such data is not committed in the SEER database.

## Conclusion

Research results based on the SEER database show that NACT may be a better treatment option than NARCT for patients with non-metastatic pancreatic cancer. However, this finding should be treated with caution because the study was based on the TNM staging system rather than clinical staging based on surgical resectability. This difference may bias the study results. For example, those who receive NARCT may include more cases with localized disease that is difficult to remove. therefore, in order to develop individualized radiotherapy regimens, more prospective randomized controlled studies are needed to compare and select different neoadjuvant therapy strategies for pancreatic cancer.

## Data availability statement

Publicly available datasets were analyzed in this study. This data can be found here: https://seer.cancer.gov/data/access.html, SEER Research Plus Dataset, 17 Registries (2000–2019).

## Author contributions

JY: Conceptualization, Methodology, Software, Validation, Visualization, Writing – original draft, Writing – review & editing. XQ: Conceptualization, Data curation, Software, Validation, Visualization, Writing – review & editing. FJ: Methodology, Supervision, Validation, Writing – review & editing. H-MQ: Data curation, Formal analysis, Investigation, Writing – review & editing. JZ: Data curation, Formal analysis, Investigation, Writing – review & editing. J-RZ: Conceptualization, Data curation, Writing – review & editing. L-JY: Conceptualization, Visualization, Writing – review & editing. A-JZ: Investigation, Project administration, Supervision, Writing – review & editing. PN: Software, Supervision, Validation, Visualization, Writing – review & editing.
